# Supplementary figures and images for: An innovative strategy to identify new targets for delivering antibodies to the brain has led to the exploration of the integrin family
Source: PLoS One. 2022 Sep 15;17(9):e0274667. doi: 10.1371/journal.pone.0274667 (PMC9477330; doi:10.1371/journal.pone.0274667)

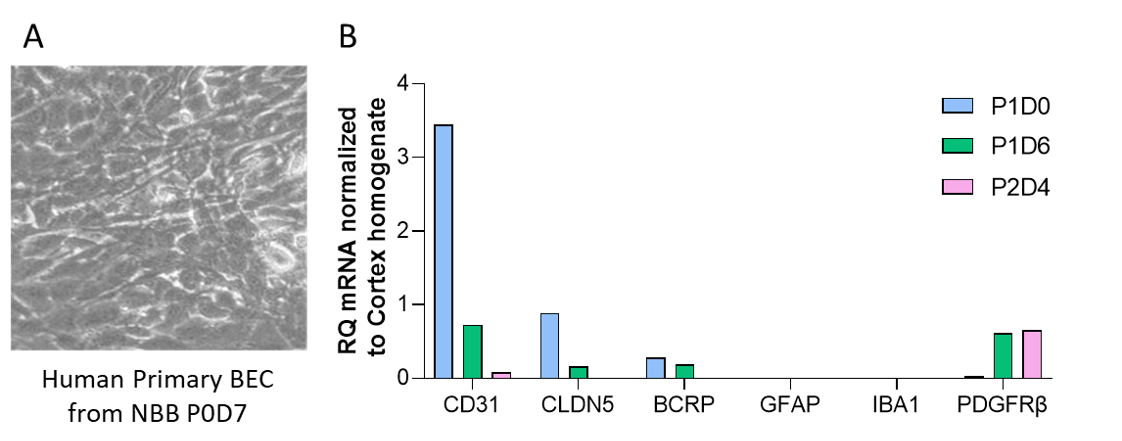

Supplement: S1 Fig — [A] Human primary brain cells isolated from NBB autopsy cases at passage 0 after 7 days in culture. Cells were observed 100X magnification with inverted microscope [B] Gene Relative Quantity [RQ] in human primary BMECs characterized by qPCR. Data were normalized to data from cortex homogenate. (TIF) [file pone.0274667.s001.tif]

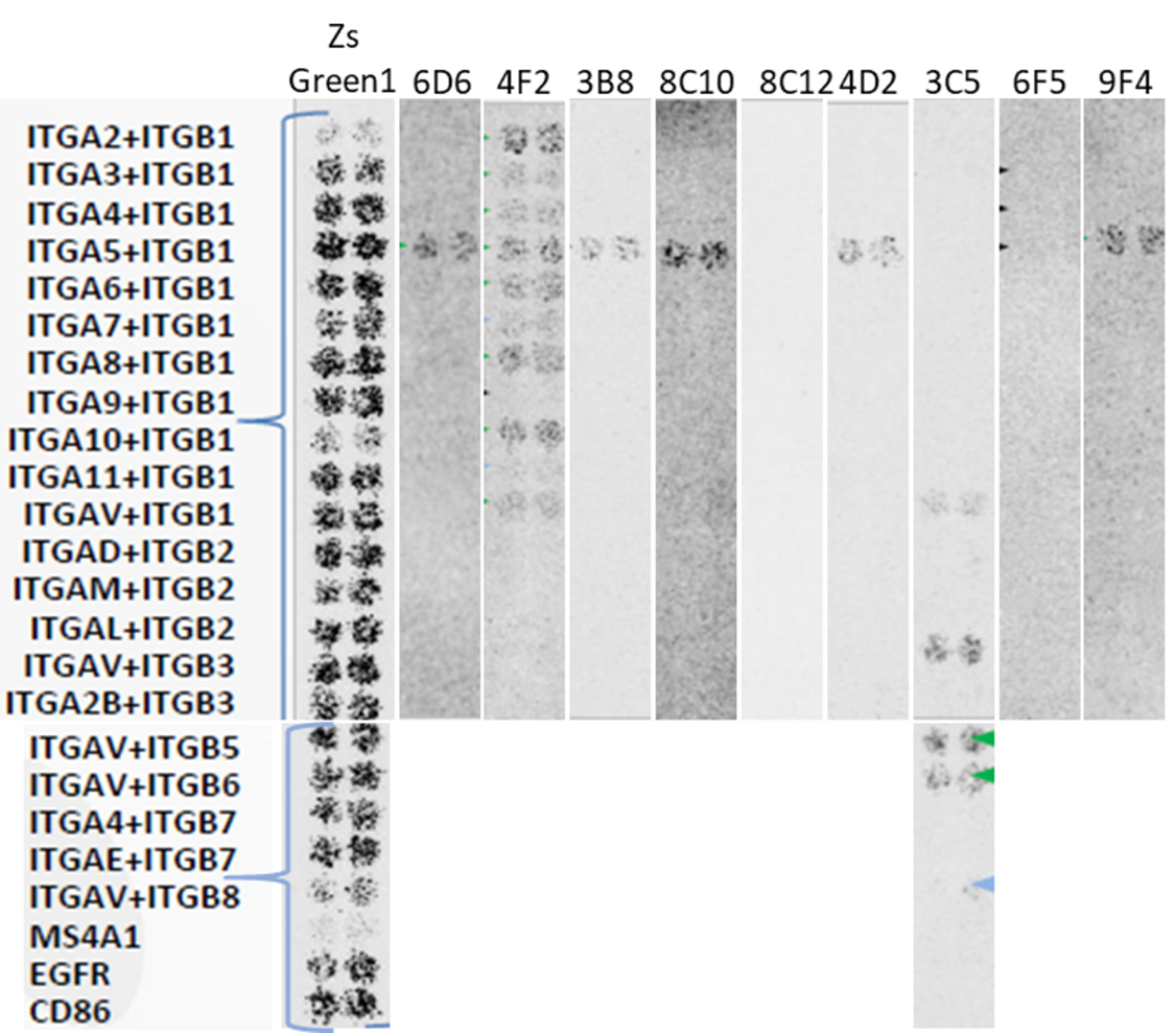

Supplement: S2 Fig — Antibodies were loaded at 5μg/mL and bound to different integrin heterodimers spotted on a slide. The signal was detected by HRP coloration. (TIF) [file pone.0274667.s002.tif]

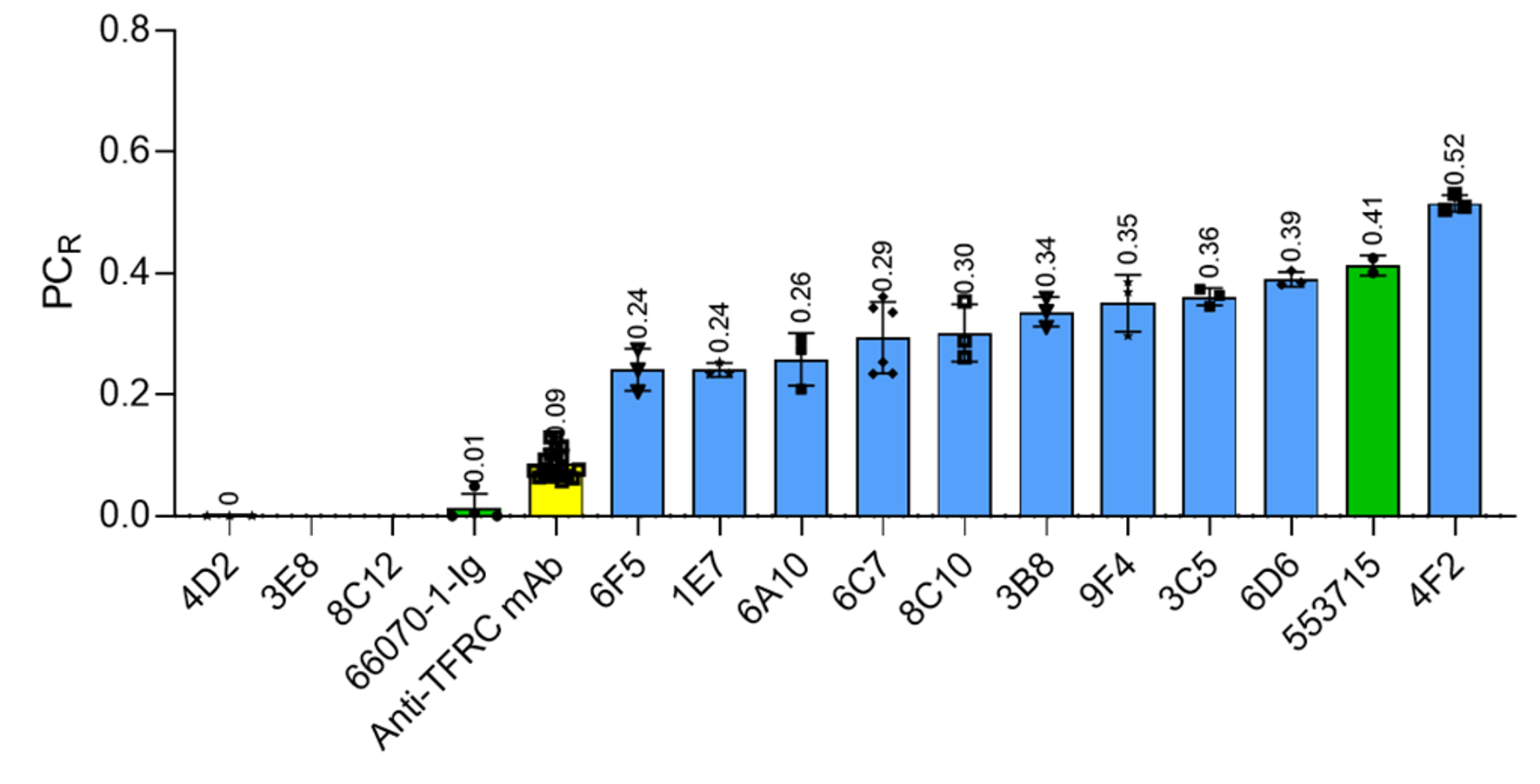

Supplement: S3 Fig — Internal anti-integrin antibodies are represented with blue bars, commercial anti-integrin antibodies are represented with green bars, anti-TFRC antibody is represented with yellow bar. Each point represents one Transwell®. (TIF) [file pone.0274667.s003.tif]

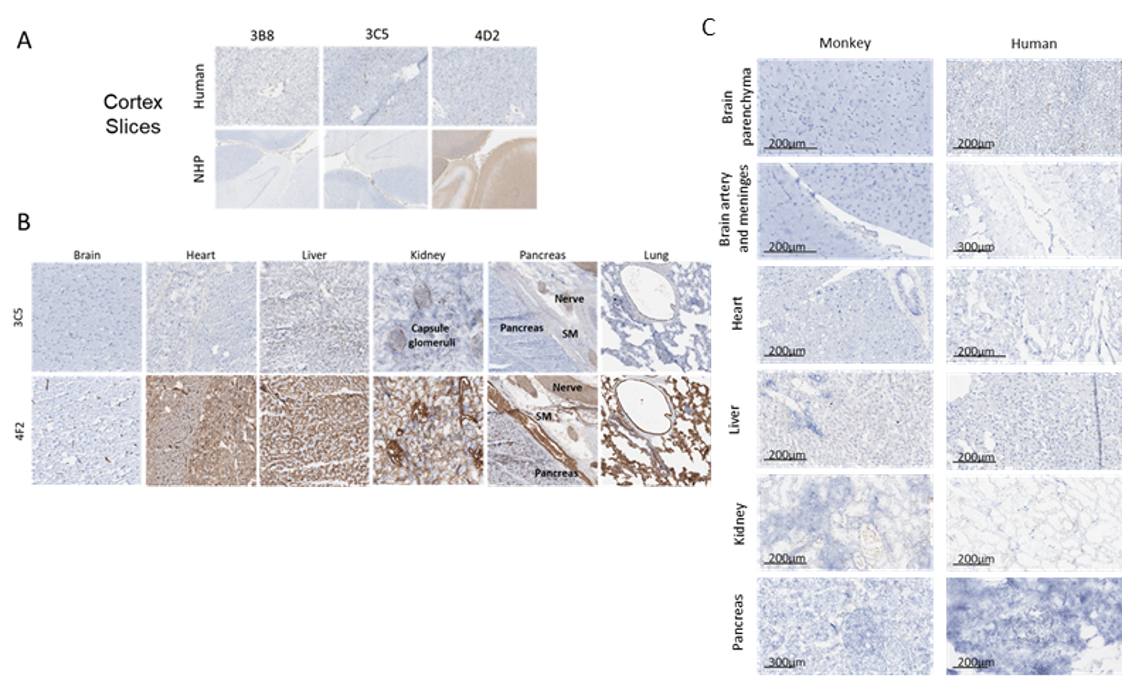

Supplement: S4 Fig — Immunostaining was done with DABMap™ chromogenic detection kit where brown color is a binding signal. A: human and NHP frozen cortex slides incubated with three different anti-integrin antibodies B: human frozen brain and peripheral tissue slices incubated with 3C5 or 4F2. C: Isotype control of monkey and human slices from Fig 5. Blue color corresponds to the hematoxylin counterstaining to visualize the cells and their nuclei. (TIF) [file pone.0274667.s004.tif]

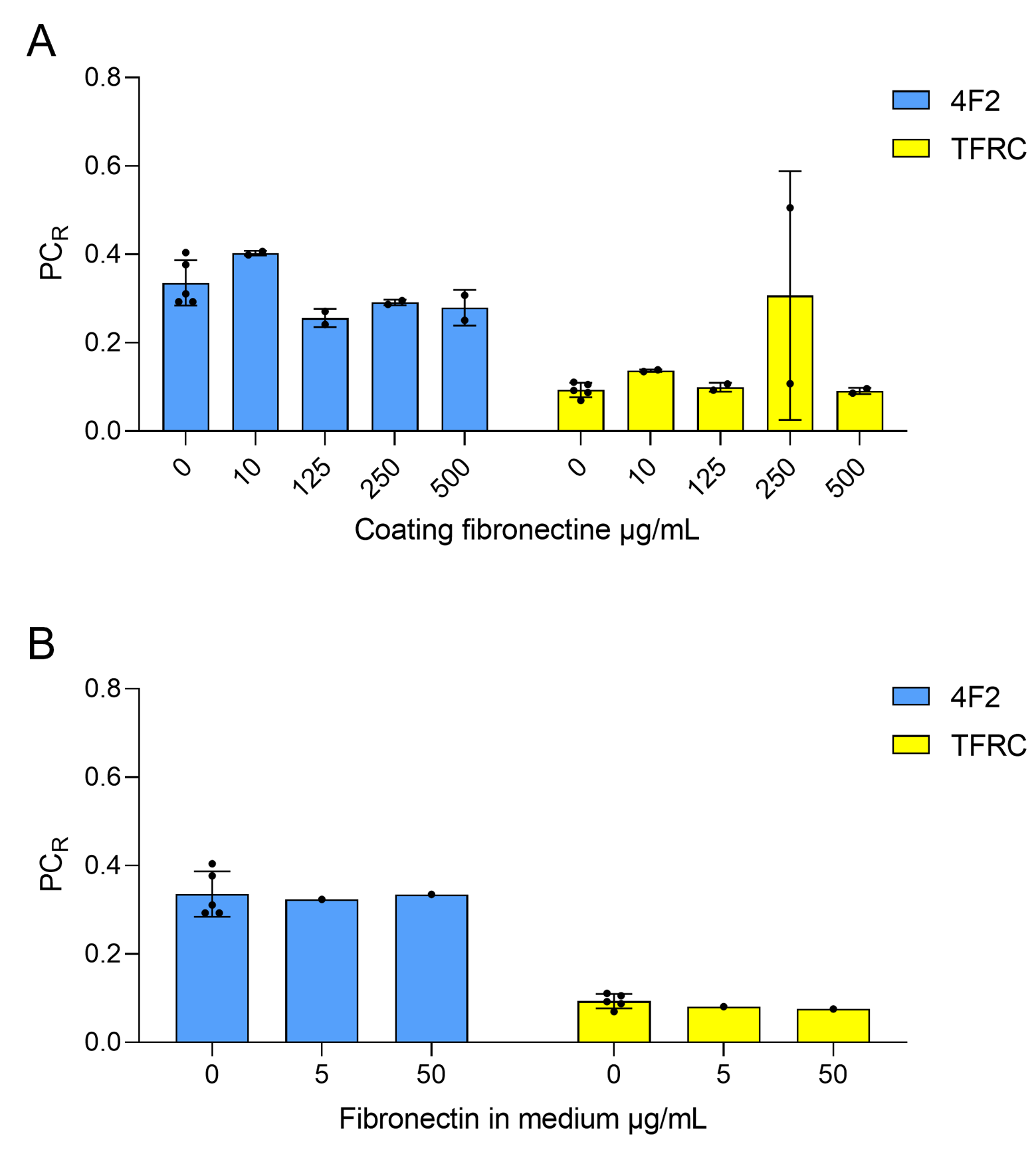

Supplement: S5 Fig — 4F2 is represented with blue bars and anti-TFRC is represented with yellow bars. A: different concentrations of fibronectin were coated on the Transwell®. B: different concentrations of fibronectin were added to the culture medium. Each point represents one Transwell®. (TIF) [file pone.0274667.s005.tif]

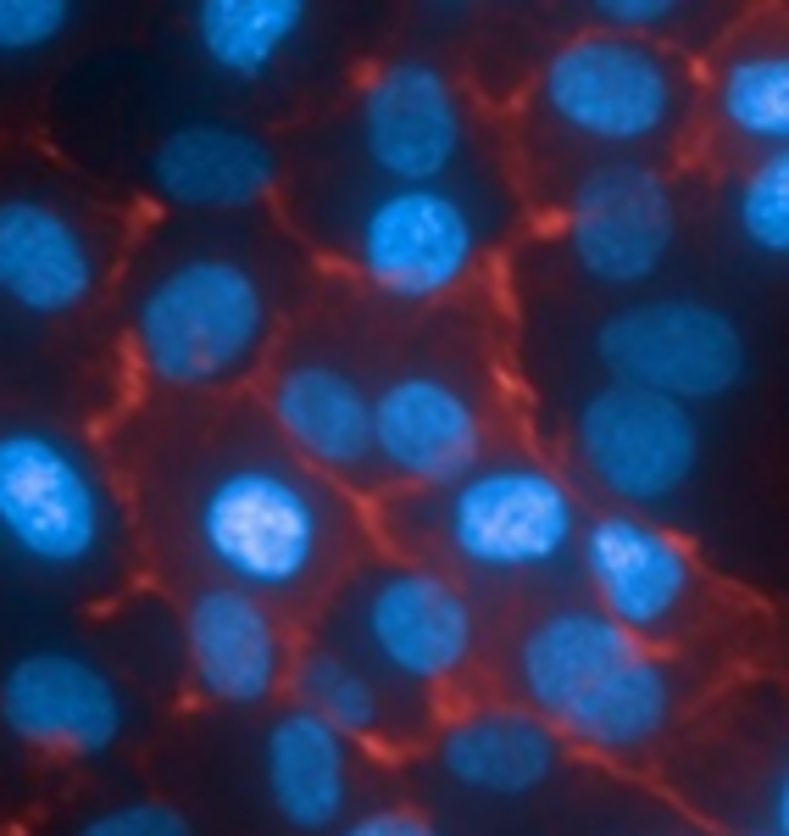

Supplement: S6 Fig — 4F2 target cellular localization was visualized by fluorescence microscopy at 200X in hCMEC/D3. Cultured cells were fixed in 4% paraformaldehyde for 15 min, at RT, and blocked in Odyssey LiCor Blocking Buffer.4F2 antibody was incubated overnight at 4°C and anti-human secondary antibody conjugated with Alexa 594 nm and Hoechst 33432 for nuclei staining was subsequently used for 2h at RT. 4F2 target is visualized in red and nuclei in blue. (TIF) [file pone.0274667.s006.tif]
