# Supplementary material for: An innovative strategy to identify new targets for delivering antibodies to the brain has led to the exploration of the integrin family
Source: PLoS One. 2022 Sep 15;17(9):e0274667. doi: 10.1371/journal.pone.0274667 (PMC9477330; doi:10.1371/journal.pone.0274667)
Supplement: S1 Table — (DOCX) [file pone.0274667.s007.docx]

|  |  | | | | **95% CI** | |  | |
| --- | --- | --- | --- | --- | --- | --- | --- | --- |
| **Parameter** | | **Antibody** | **N** | **Mean** | **Lower** | **Upper** | **p-value** | **Adj. p-value** |
| **PCR in NHP BMECs** | | Anti-TFRC mAb | 3 | 0.326 | 0.210 | 0.442 | 0.0070 | **0.0210** |
|  | | 1E7 | 3 | 0.241 | 0.186 | 0.295 | 0.0040 | **0.0183** |
|  | | 3B8 | 3 | 0.353 | 0.292 | 0.415 | 0.0016 | **0.0112** |
|  | | 3C5 | 3 | 0.339 | 0.288 | 0.390 | 0.0012 | **0.0098** |
|  | | 4D2 | 3 | 0.250 | 0.224 | 0.276 | 0.0008 | **0.0073** |
|  | | 4F2 | 9 | 0.457 | 0.429 | 0.485 | <.0001 | **<.0001** |
|  | | 6A10 | 3 | 0.208 | 0.094 | 0.322 | 0.0275 | 0.0550 |
|  | | 6C7 | 3 | 0.081 | 0.021 | 0.141 | 0.8460 | 0.8460 |
|  | | 8C10 | 3 | 0.254 | 0.197 | 0.311 | 0.0037 | **0.0183** |
|  | | 8C12 | 3 | 0.442 | 0.355 | 0.528 | 0.0017 | **0.0112** |
| **PCR in hCMEC/cells** | | Anti-TFRC mAb | 18 | 0.086 | 0.074 | 0.098 | 0.9896 | 0.9896 |
|  | | 1E7 | 3 | 0.241 | 0.213 | 0.269 | 0.0011 | **0.0074** |
|  | | 3B8 | 3 | 0.337 | 0.277 | 0.396 | 0.0017 | **0.0103** |
|  | | 3C5 | 3 | 0.361 | 0.326 | 0.397 | 0.0005 | **0.0039** |
|  | | 4F2 | 3 | 0.515 | 0.481 | 0.550 | 0.0002 | **0.0020** |
|  | | 6A10 | 3 | 0.258 | 0.150 | 0.367 | 0.0122 | 0.0357 |
|  | | 6C7 | 6 | 0.294 | 0.232 | 0.356 | 0.0002 | **0.0024** |
|  | | 6D6 | 3 | 0.390 | 0.361 | 0.420 | 0.0003 | **0.0025** |
|  | | 6F5 | 3 | 0.241 | 0.155 | 0.327 | 0.0097 | 0.0357 |
|  | | 8C10 | 3 | 0.302 | 0.184 | 0.419 | 0.0089 | 0.0357 |
|  | | 9F4 | 3 | 0.351 | 0.233 | 0.468 | 0.0058 | 0.0291 |
|  | Adjusted P-values using Bonferonni Holm correction Significant ajusted p-values at 2.5% level are highlighted in bold | | | | | | | |

**S1 Table: Statistical results for pulse chase ratio**
