# Supplementary material for: An innovative strategy to identify new targets for delivering antibodies to the brain has led to the exploration of the integrin family
Source: PLoS One. 2022 Sep 15;17(9):e0274667. doi: 10.1371/journal.pone.0274667 (PMC9477330; doi:10.1371/journal.pone.0274667)
Supplement: S2 Table — (DOCX) [file pone.0274667.s008.docx]

|  |  | **95% CI** | | | | | **Comparison versus NC** | | | |  |  |
| --- | --- | --- | --- | --- | --- | --- | --- | --- | --- | --- | --- | --- |
| **Parameter** | **Antibody comparison vs NC** | **N** |  |  | **Harmonic mean** | **Lower** | | **Upper** | **p-value** | **Dunnett**  **adj. p-value** | | |
| **Papp test antibody/control antibody** | NC | 3 |  |  | 1.056 | 0.980 | | 1.145 | - | **-** | | |
|  | Anti-TFRC mAb | 6 |  |  | 11.713 | 6.883 | | 39.276 | <.0001 | **<.0001** | | |
|  | 9F4 | 4 |  |  | 5.266 | 3.799 | | 8.584 | <.0001 | **<.0001** | | |
|  | 8C10 | 4 |  |  | 3.707 | 2.914 | | 5.092 | <.0001 | **<.0001** | | |
|  | 6D6 | 4 |  |  | 3.991 | 3.087 | | 5.643 | <.0001 | **<.0001** | | |
|  | 6C7 | 3 |  |  | 4.685 | 3.354 | | 7.769 | <.0001 | **<.0001** | | |
|  | 4F2 | 5 |  |  | 8.735 | 5.552 | | 20.473 | <.0001 | **<.0001** | | |
|  | 4D2 | 4 |  |  | 6.138 | 4.232 | | 11.167 | <.0001 | **<.0001** | | |
|  | 3E8 | 4 |  |  | 1.135 | 1.047 | | 1.238 | 0.0735 | 0.3023 | | |
|  | 3B8 | 4 |  |  | 5.773 | 4.055 | | 10.017 | <.0001 | **<.0001** | | |
|  |  |  | Inverse transformation was applied to the data before analysis Significant ajusted p-values at 2.5% level are highlighted in bold | | | | | | | | |  |

**S2 Table: Statistical results for transcytosis ratio**
