# Supplementary material for: An innovative strategy to identify new targets for delivering antibodies to the brain has led to the exploration of the integrin family
Source: PLoS One. 2022 Sep 15;17(9):e0274667. doi: 10.1371/journal.pone.0274667 (PMC9477330; doi:10.1371/journal.pone.0274667)
Supplement: S3 Table — (DOCX) [file pone.0274667.s009.docx]

| **clone** | **proteine pI** | **HC pI** | **LC pI** |
| --- | --- | --- | --- |
| **4F2** | 7,1 | 7,9 | 5,3 |
| **6F5** | 8,05 | 8,3 | 7,1 |
| **8C10** | 7,95 | 8,2 | 7,1 |
| **3B8** | 8,15 | 8,3 | 7,7 |
| **3C5** | 7,95 | 8,05 | 7,7 |
| **4D2** | 7,85 | 7,9 | 7,7 |
| **6D6** | 7,85 | 8,3 | 5,9 |
| **8C12** | 7,7 | 8,05 | 6,5 |
| **9F4** | 7,85 | 8,2 | 6,5 |

**S3 Table: Total protein, Heavy chain and Light chain pI calculated for test antibodies**
