# Supplementary material for: An innovative strategy to identify new targets for delivering antibodies to the brain has led to the exploration of the integrin family
Source: PLoS One. 2022 Sep 15;17(9):e0274667. doi: 10.1371/journal.pone.0274667 (PMC9477330; doi:10.1371/journal.pone.0274667)
Supplement: Attachment — Submitted filename: Response to reviewers.docx [file pone.0274667.s011.docx]

Reviewer #2: The authors responded well to all of the comments and suggestions made. This manuscript represents an extremely thorough attempt to screen for an identify antibodies for BBB targeting and transcytosis. The combination of in vitro assays was impressive and is as convincing as can be reasonable expected. The remaining comments are minor and are related to some polishing and clarification.

The pulse chase assay was performed with cells with TEER below 150 ohm cm2, but the transcytosis assay was described as using cells with a high TEER but is also described as having at TEER up to 150 ohm cm2. Was there a minimum TEER value considered in either situation? Was there a difference in the NHP cells selected for the two different techniques? This should be explicitly state in either the results narrative or the materials methods.

In fact, it was an error, so line 367, we have replaced “up to 150 Ω.cm^2^” by “from 150 Ω.cm^2^”.

Citation(s) is needed on line 65 outlining the additional identified receptors.

The paragraph lines 71-81 is almost directly duplicated in the next paragraph. All of this is good information to back up and ground this manuscript, but it would benefit from some polishing.
To address the two comments above we have reworked the introduction lines 70,71, 80,81 and 84.

Figure 2 – it would improve readability if the bars were ordered the same in C as they are in B and E

We thank the reviewer for this observation, and we have corrected the Figure 2.
